# New opportunities and challenges for nonshedder tumors

**DOI:** 10.1016/j.fmre.2025.12.002

**Published:** 2025-12-05

**Authors:** Zifan Li, Yue He, Jing Bai, Hongke Wang, Kezhong Chen

**Affiliations:** a Department of Thoracic Surgery, Peking University People’s Hospital, Beijing 100044, China; b Thoracic Oncology Institute, Peking University People’s Hospital, Beijing 100044, China; c Research Unit of Intelligence Diagnosis and Treatment in Early Non-small Cell Lung Cancer, Chinese Academy of Medical Sciences (2021RU002) Peking University People’s Hospital, Beijing 100044, China; d Institute of Advanced Clinical Medicine, Peking University, Beijing 100191, China; e College of Future Technology, Peking University, Beijing 100871, China; f Peking University Medical Industrial Park, Zhongguancun Life Science Park, Geneplus-Beijing Institute, Beijing 102206, China

**Keywords:** Neoplasms, Circulating tumor DNA, Liquid biopsy, Recurrence, Nonshedder

## Abstract

Cancer remains a leading cause of mortality worldwide. While surgical resection effectively controls localized tumors, postoperative recurrence remains a significant challenge. Circulating tumor DNA (ctDNA), a key biomarker in liquid biopsy, has garnered considerable attention for its non-invasive nature and high specificity in monitoring cancer recurrence. However, some patients exhibit ctDNA nonshedding characteristics before surgery, and the underlying mechanisms remain unclear. This review examines the distinctive features of nonshedder tumors, highlighting the influence of tumor stage, metabolic activity, genetic variation, and the tumor microenvironment on ctDNA shedding. Based on these insights, we discuss emerging opportunities and challenges associated with nonshedder tumors and propose directions for future research.

## 1 Introduction

Cancer remains a leading cause of mortality worldwide, posing a significant public health challenge and imposing a substantial burden on healthcare systems. Surgical resection is a primary treatment modality for tumors and is effective in controlling localized disease. However, postoperative recurrence remains a major concern. Consequently, early identification of high-risk populations and timely interventions are crucial for improving patient prognosis. In recent years, liquid biopsy technologies have emerged as valuable tools for cancer recurrence monitoring, with circulating tumor DNA (ctDNA) attracting particular attention due to its high specificity. ctDNA comprises DNA fragments released into the bloodstream by tumor cells through apoptosis, necrosis, or active secretion [1]. These fragments contain tumor-specific genetic alterations, enabling real-time monitoring of tumor molecular characteristics and dynamic changes. With advances in ctDNA detection, ctDNA-based minimal residual disease (MRD) assessment has become an increasingly important approach for monitoring cancer recurrence. Current expert consensus guidelines indicate that ctDNA detection performed at baseline (pre-treatment), landmark (within 1 month post-treatment), and longitudinal timepoints (every 3–6 months) carries significant prognostic value in cancers such as colorectal cancer, non-small cell lung cancer, and breast cancer [2,3]. Patients with positive ctDNA status exhibit significantly worse prognosis compared to those with negative ctDNA. Furthermore, with respect to guiding adaptive treatment strategies, available evidence suggests that ctDNA- patients may be considered for de-escalation therapy, whereas ctDNA+ patients may benefit from escalation therapy. Nonetheless, additional randomized controlled trials are warranted to confirm these findings [2,3]. ctDNA shedding refers to the collective process by which tumor-derived DNA enters the bloodstream, with its levels influenced by generation, release, and clearance dynamics [4]. Nonshedder tumors are defined as those in which pretreatment ctDNA levels are undetectable by current assays, regardless of tumor stage or pathological subtype [[5], [6], [7], [8], [9]]. As early as 2022, the European Society for Medical Oncology (ESMO) highlighted that “more research is needed to understand the molecular basis of ctDNA shedding into blood” [10]. However, the mechanisms underlying ctDNA nonshedding remain unclear. Moreover, while studies suggest that nonshedder tumors are generally associated with better prognosis compared to shedder tumors, a significant proportion of these patients still experience postoperative recurrence [11]. Through multi-omics analysis of nonshedder tumor molecular mechanisms and advancements in detection technologies, precise preoperative prognosis stratification may be achieved. This could provide earlier and more reliable evidence for perioperative management, recurrence monitoring, and personalized cancer treatment.

## 2 Principles of current ctDNA detection technologies

Current mainstream ctDNA detection technologies primarily utilize next-generation sequencing (NGS) to identify ctDNA mutations in blood samples. These approaches can be broadly categorized into tumor-agnostic and tumor-informed methods based on their requirement for prior tumor mutation information [12].

The tumor-agnostic approach utilizes standardized ctDNA detection panels constructed by selecting mutation sites strongly associated with tumorigenesis and progression from existing genomic databases, as exemplified by CAPP-Seq [13]. In contrast, tumor-informed strategies comprise both fixed and personalized panels. Tumor-informed fixed panels (e.g., MinerVA [14]) employ comprehensive sequencing panels to initially characterize tumor tissue mutations, followed by ctDNA analysis using the same panel; only tumor-derived mutations detected in ctDNA are reported as positive. Meanwhile, tumor-informed personalized panels (such as Signatera [15] and RaDaR [16]) involve whole-exome sequencing of tumor tissue to identify patient-specific genomic alterations, based on which customized panels are designed for subsequent ctDNA monitoring. It should be noted that somatic mutations and clonal hematopoiesis in peripheral blood may contribute biological noise to ctDNA detection. Current best practice recommends matched white blood cell sequencing for noise filtering to reduce false-positive results [10,17].

Overall, our study demonstrates that both tumor-agnostic and tumor-informed strategies generally exhibit high specificity [5]. The tumor-agnostic approach offers distinct advantages in terms of a more standardized workflow, faster turnaround time, and lower cost, although its sensitivity requires further improvement. In contrast, the tumor-informed methods, particularly the tumor-informed personalized approach, achieve significantly higher sensitivity. Moving forward, it will be crucial to reduce the turnaround time and cost of tumor-informed methods while maintaining their high sensitivity. Nevertheless, the diversity of available technologies also increases the complexity of result interpretation. To facilitate accurate clinical assessment of ctDNA reports and disease risk, the European Liquid Biopsy Society recommends that ctDNA detection reports clearly document the detection method (e.g., panel name and technical platform), the range of genes covered, key performance characteristics, and quality control parameters—such as sequencing depth, DNA extraction efficiency, and number of input molecules [18].

## 3 Characteristics of nonshedder tumors

**Fig. 1:**
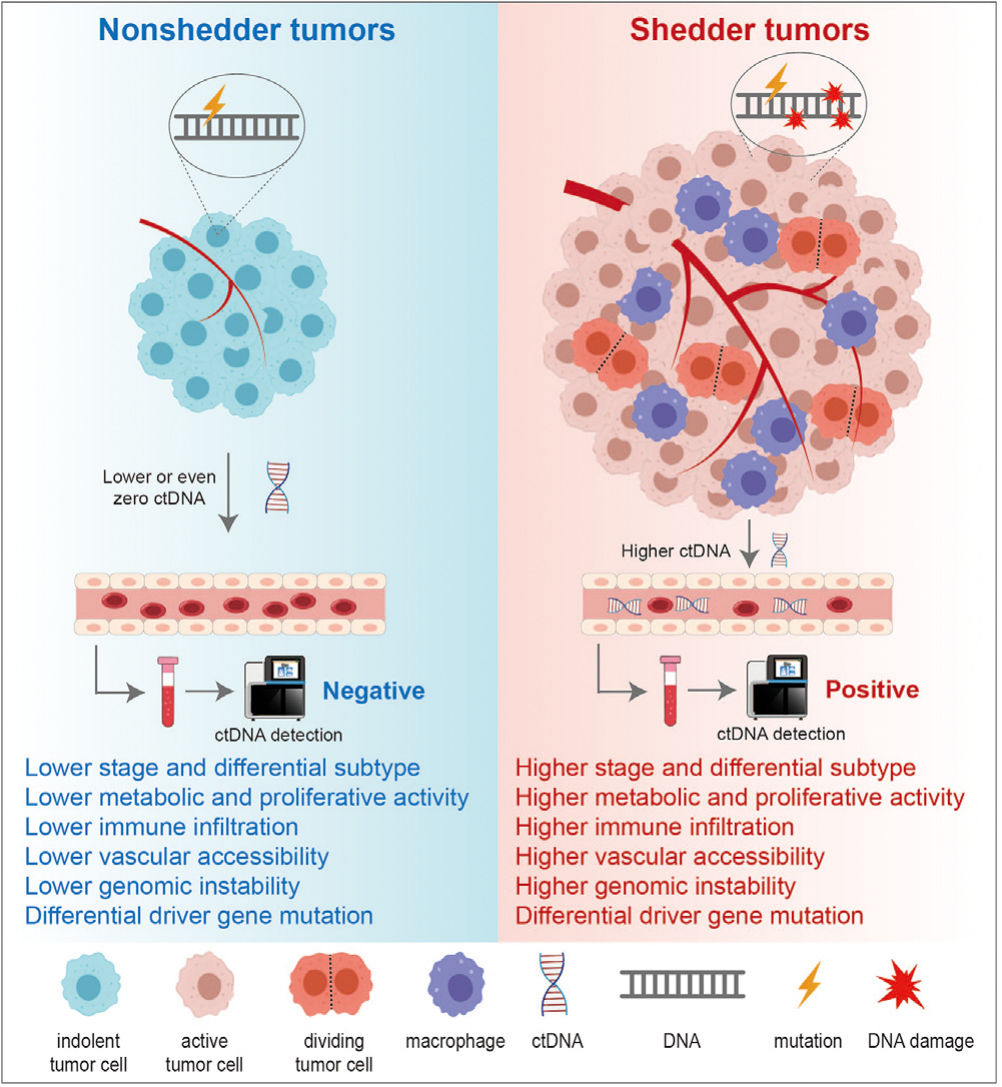
**Comparison of characteristics between nonshedder tumors and shedder tumors.** Current evidence suggests that tumors at earlier stages, with lower activity, reduced immune infiltration, and greater genomic stability, are more likely to exhibit ctDNA nonshedding. Additionally, certain pathological subtypes and driver gene mutations are significantly associated with nonshedders.

By systematically comparing clinical, imaging, pathological, and genetic data from patients with shedder and nonshedder tumors, a deeper understanding of the unique clinical and molecular characteristics of nonshedder tumors can be achieved. This section comprehensively summarizes the key features of nonshedder tumors across various domains (Fig. 1) and highlights critical scientific questions that remain unresolved, with the aim of providing direction for future in-depth investigations.

### 3.1 Tumor stage and subtype

Early-stage tumors, due to their small size and lower tumor burden, may release ctDNA at concentrations below the detection limits of current technologies, resulting in the nonshedder tumor phenotype [19]. Moreover, significant variations in ctDNA shedding potential exist across different pathological subtypes. For instance, in lung cancer, nonshedder tumors are predominantly adenocarcinoma [20]; in urothelial carcinoma, the basal/squamous subtype is significantly enriched in shedder tumors [9]; and in breast cancer, human epidermal growth factor receptor 2 (HER2)-positive and triple-negative breast cancer (TNBC) subtypes exhibit markedly higher ctDNA release levels compared to luminal-type carcinomas [21]. These observations suggest that distinct pathological subtypes may possess unique biological characteristics that influence ctDNA release dynamics.

### 3.2 Tumor metabolism and proliferative activity

There are significant differences in metabolic activity across various tumors, which also impact the release of ctDNA. For instance, in lung cancer patients undergoing PET-CT scans, preoperative ctDNA concentrations correlate strongly with the tumor’s maximum standardized uptake value (SUVmax), indicating that metabolically active tumors tend to release more ctDNA [6].

The rate of tumor cell proliferation is another key factor influencing ctDNA release. Studies have shown that tumors with higher proliferation rates generally exhibit higher rates of apoptosis and necrosis, resulting in more ctDNA being released into the bloodstream. In contrast, slower-growing tumors with less apoptosis and necrosis tend to release relatively lower amounts of ctDNA. This phenomenon has been validated in multiple tumor types. For instance, gene enrichment analysis in colorectal cancer, lung cancer, urothelial carcinoma, and breast cancer has revealed that shedder tumors are often enriched with genes associated with proliferation and cell cycle regulation, further confirming the close relationship between proliferative activity and ctDNA release [7,8,20,22].

### 3.3 Tumor microenvironment (TME)

Tumors consist of both tumor cells and their surrounding microenvironment, which includes various cellular components and non-cellular substances, such as supporting cells, the extracellular matrix (ECM), blood vessels, immune cells, and a range of signaling molecules [23]. The components of the microenvironment play a crucial role in ctDNA shedding. For instance, macrophages can phagocytose necrotic tumor cells and release the digested ctDNA into the bloodstream, suggesting that the degree of macrophage infiltration in the tumor may be associated with ctDNA shedding [1]. Moreover, in breast cancer, researchers have identified immune response pathways related to ctDNA release through Leading-Edge Analysis (LEA). They found that the IL-6/JAK/STAT3 pathway and NF-κB pathway were enriched in shedder tumors, whereas the TGF-β pathway was enriched in nonshedder tumors [7]. Mechanistically, we found that the TGF-β pathway may suppress cell proliferation and immune infiltration, potentially contributing to reduced ctDNA release [24]. However, both the IL-6/JAK/STAT3 pathway and NF-κB signaling demonstrate complex, context-dependent pleiotropic functions encompassing inflammation activation, anti-apoptotic effects, and genomic stability modulation [25,26]. Current understanding remains primarily phenomenological, and the precise mechanistic links between IL-6/JAK/STAT3 or NF-κB activation and enhanced ctDNA release remain to be determined. Further experimental investigations are required to clarify these relationships.

Since ctDNA detection typically relies on blood samples, the degree of vascular infiltration in tumors significantly impacts the levels of ctDNA in the bloodstream. Generally, tumors with higher vascular infiltration are more likely to release ctDNA into circulation [19]. Additionally, the endothelial barrier of blood vessels plays an important regulatory role in the process of ctDNA entry into the bloodstream. The vascular permeability can vary significantly across different tumors and even within different regions of the same tumor, directly influencing ctDNA release. For example, some tumors may exhibit low vascular permeability due to high expression of tight junction proteins between endothelial cells or stronger integrity of the basement membrane, which restricts ctDNA release. However, research on vascular accessibility remains limited, and further exploration is needed in the future.

### 3.4 Tumor variation

Firstly, nonshedder tumors generally exhibit lower levels of genomic instability. For example, in lung cancer, nonshedder tumors have lower tumor mutational burden, heterozygous deletions, and whole-genome duplication compared to shedder tumors [5,20]. In colorectal cancer, tumors with microsatellite instability release higher levels of ctDNA [8].

Furthermore, a close relationship exists between tumor driver gene mutations and ctDNA shedding. Studies have revealed that high-frequency TP53 mutations promote ctDNA release in lung cancer, urothelial carcinoma, and appendiceal adenocarcinoma [5,9,27]. Mechanistic investigations suggest that TP53 mutations may contribute to this phenomenon through multiple pathways [28]: (1) by inducing genomic instability that leads to chromosomal aberrations and mutation accumulation, thereby increasing DNA fragmentation and subsequent ctDNA release; (2) through disruption of cell cycle control, allowing proliferation of cells with DNA damage; and (3) via modulation of the tumor microenvironment, particularly through effects on angiogenesis and immune suppression, which may indirectly influence ctDNA release and clearance. Conversely, high-frequency KRAS mutations in colorectal cancer appear to suppress ctDNA release [8]. Mechanistically, KRAS mutations are predominantly enriched in the metabolically adapted CMS3 subtype and are associated with TGF-β signaling, suggesting that KRAS-mutant CMS3 tumors may establish an immunosuppressive microenvironment that inhibits ctDNA shedding [29]. However, it should be noted that the current evidence remains incomplete, and these proposed mechanisms require further experimental validation.

Additionally, emerging data implicate other driver gene mutations (e.g., EGFR, MLL2/3, ATM, FAT1, and SMARCA4) in modulating ctDNA release patterns. Nevertheless, the clinical significance and reproducibility of these observations warrant more extensive investigation through well-designed prospective studies [6,19].

## 4 Prospects in nonshedder tumors

### 4.1 Investigating the mechanisms of ctDNA nonshedding

**Fig. 2:**
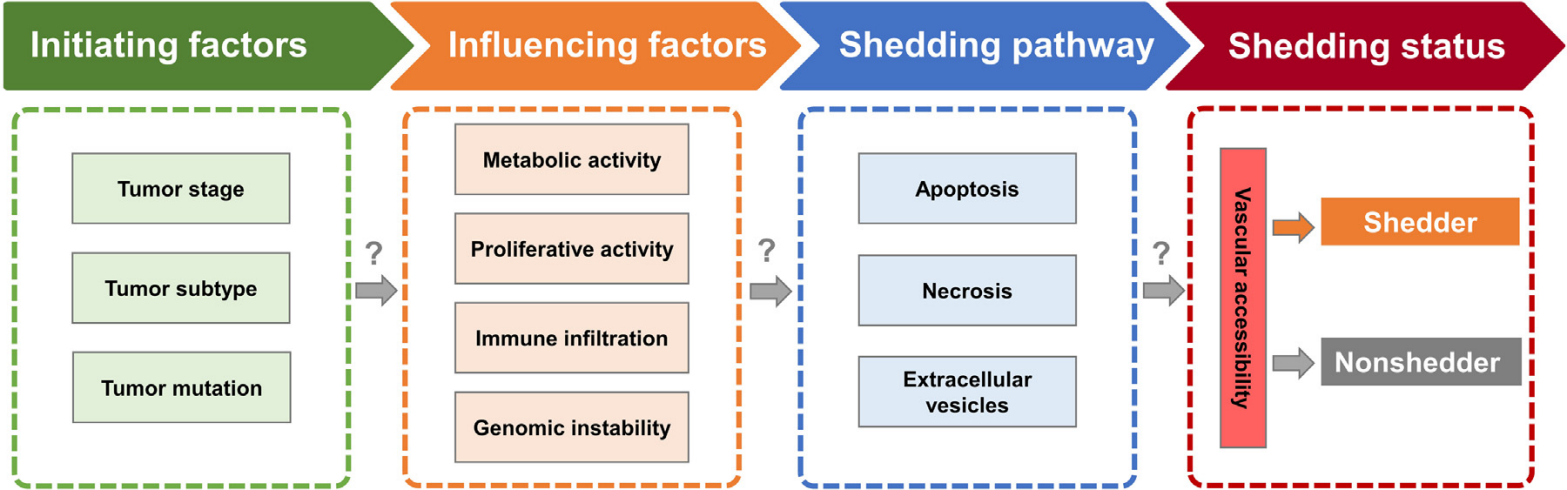
**Proposed regulatory framework for ctDNA release.** Driver mutations, tumor stage and subtype modulate proliferation, metabolism, immune activity and genomic stability to determine ctDNA shedding, with vascular accessibility controlling subsequent circulation entry.

Although existing studies have preliminarily revealed the clinical and molecular characteristics of nonshedder tumors, the relationships between these features remain unclear. As summarized earlier, potential factors influencing ctDNA release include tumor stage/subtype/activity, the tumor microenvironment, and tumor variation. Additionally, research indicates that ctDNA is primarily released into the bloodstream via three pathways: (1) apoptosis, (2) necrosis, and (3) extracellular vesicle secretion [1]. However, the pathway by which these potential factors affect ctDNA entry into the blood remains unknown. Here, we propose a potential regulatory framework (Fig. 2): driver mutations, tumor stage and subtype act as initiating factors, modulating tumor proliferation, metabolism, immune activity, and genomic stability, which in turn influence ctDNA release, while vascular accessibility determines whether released ctDNA successfully enters circulation.

To address this knowledge gap, leveraging advanced multi-omics platforms (e.g., radiomics, single-cell omics, spatial omics) will provide powerful tools for systematically characterizing nonshedder tumors across multiple dimensions. By focusing on key features identified through screening, future studies could further elucidate the molecular mechanisms and signaling pathways regulating ctDNA release. For instance, investigating tumor cell resistance to apoptosis/necrosis, dynamic changes in extracellular vesicle secretion, or immune-tumor cell interactions in the TME may provide critical insights into ctDNA shedding regulation. This process further involves the identification of specific genes or transcription factors implicated in ctDNA release. Ultimately, integrating gene-edited animal models (e.g., CRISPR-Cas9) to validate key regulatory factors could help establish a comprehensive mechanistic link between nonshedder tumor traits and ctDNA release suppression.

### 4.2 Strategies to enhance the sensitivity of ctDNA detection

Recent studies have demonstrated that even ultralow levels of plasma ctDNA (< 80 ppm, below the detection threshold of most current assays) remain prognostic for poor outcomes in cancer patients after curative surgery [30]. This suggests that nonshedder tumors may be classified into two subtypes: (1) true nonshedders with minimal or zero ctDNA release, and (2) “lowshedders” with ctDNA levels too low for detection by existing technologies. Thus, improving detection sensitivity is critical to distinguishing these groups. Current strategies focus on the following approaches:

#### 4.2.1 Personalized panels based on whole-genome sequencing (WGS)

Existing tumor-informed ctDNA assays primarily rely on WES, which captures fewer mutations than WGS. For instance, Chen et al. developed a patient-specific ctDNA panel using WES, achieving a limit of detection (LOD) of 0.004% [5]. Further advancing this, Black et al. introduced NeXT Personal, a WGS-based tumor-informed assay with an ultrahigh sensitivity of 0.000133% [30], enabling more precise patient stratification.

#### 4.2.2 Personalized panel design with multi-site sampling

Due to high intra-tumoral heterogeneity, a single tumor often contains multiple cell clusters harboring diverse clonal and subclonal mutations [31,32]. During metastatic progression, metastases may arise from one or several tumor clones [33]. Existing personalized ctDNA panels, typically designed based on single-site biopsies, may not fully capture the complete clonal architecture of the primary tumor. This limitation could lead to undetected recurrences driven by tumor clones not represented in the panel. However, the development of personalized probe panels utilizing multi-site sampling remains unexplored and warrants further investigation in future studies.

#### 4.2.3 ctDNA methylation profiling

Aberrant DNA methylation is more pervasive than somatic mutations, making methylation-based ctDNA detection a promising approach [34]. Chen et al. identified tumor-specific methylation markers by comparing tumor and adjacent normal tissues, developing a tumor-informed methylation-based MRD model (timMRD). This model outperformed mutation-based assays in detecting postoperative recurrence, particularly in early-stage (e.g., Stage I) patients with low tumor burden [35].

#### 4.2.4 Prolonging ctDNA half-life

The clearance of ctDNA from blood is mediated by circulating nucleases, macrophage uptake in the liver/spleen, and renal excretion, resulting in a short half-life (typically 16 min to 2.5 h) [36]. A recent study reported that encapsulating ctDNA in lipid nanoparticles or conjugating it with monoclonal antibodies significantly reduced degradation, improving recovery rates [37]. Extending ctDNA half-life could thus convert some lowshedders into detectable cases, addressing current sensitivity limitations.

### 4.3 Exploring the potential of other biomarkers

#### 4.3.1 Plasma metabolomics and proteomics

Plasma metabolomics involves the detection of small-molecule metabolites (< 1200 Da), including lipids, sugars, nucleotides, and amino acids, which are highly sensitive to biological activity and pathological conditions, providing a direct and accurate reflection of systemic status [38,39]. Plasma proteomics refers to the comprehensive analysis of protein composition, abundance, modifications, and interactions [40]. Current research has primarily focused on early cancer detection, demonstrating promising pan-cancer performance [41,42]. However, few studies have explored their utility as MRD biomarkers. Investigating their prognostic relevance may hold significant clinical value.

#### 4.3.2 Cell-free RNA (cfRNA)

cfRNA comprises extracellular RNA fragments released into circulation via cellular metabolism or apoptosis. While cfRNA is a potential source for early detection biomarkers, 99% of blood-derived cfRNA originates from hematopoietic cells (e.g., platelets, leukocytes), making tumor-derived cfRNA exceptionally rare and technically challenging to detect [43]. Recently, Stanford researchers developed RARE-seq, a method combining a “rare abundance gene” (RAG) capture panel with a platelet-contamination correction algorithm, achieving > 80% sensitivity in identifying tumor-derived cfRNA across multiple cancers (e.g., lung, pancreatic, and liver) [44]. Further exploration of cfRNA’s prognostic utility may address challenges in non-shedding tumors.

#### 4.3.3 Circulating tumor cells (CTCs)

CTCs are tumor cells shed into circulation from primary or metastatic sites [45]. Emerging evidence supports their clinical value in early detection, prognosis prediction, and treatment monitoring. For instance, Dandachi et al. reported a prospective study of 40 resectable lung adenocarcinoma patients, where preoperative CTC positivity (37.5%, 15/40) independently predicted shorter disease-free survival and higher recurrence risk [46]. However, low CTC abundance in blood and high false-negative rates of current detection technologies limit their utility in non-shedding tumors [47]. Novel sensitivity-enhancing approaches are urgently needed.

### 4.4 Investigating prognostic features of nonshedder tumors and enabling personalized monitoring

**Fig. 3:**
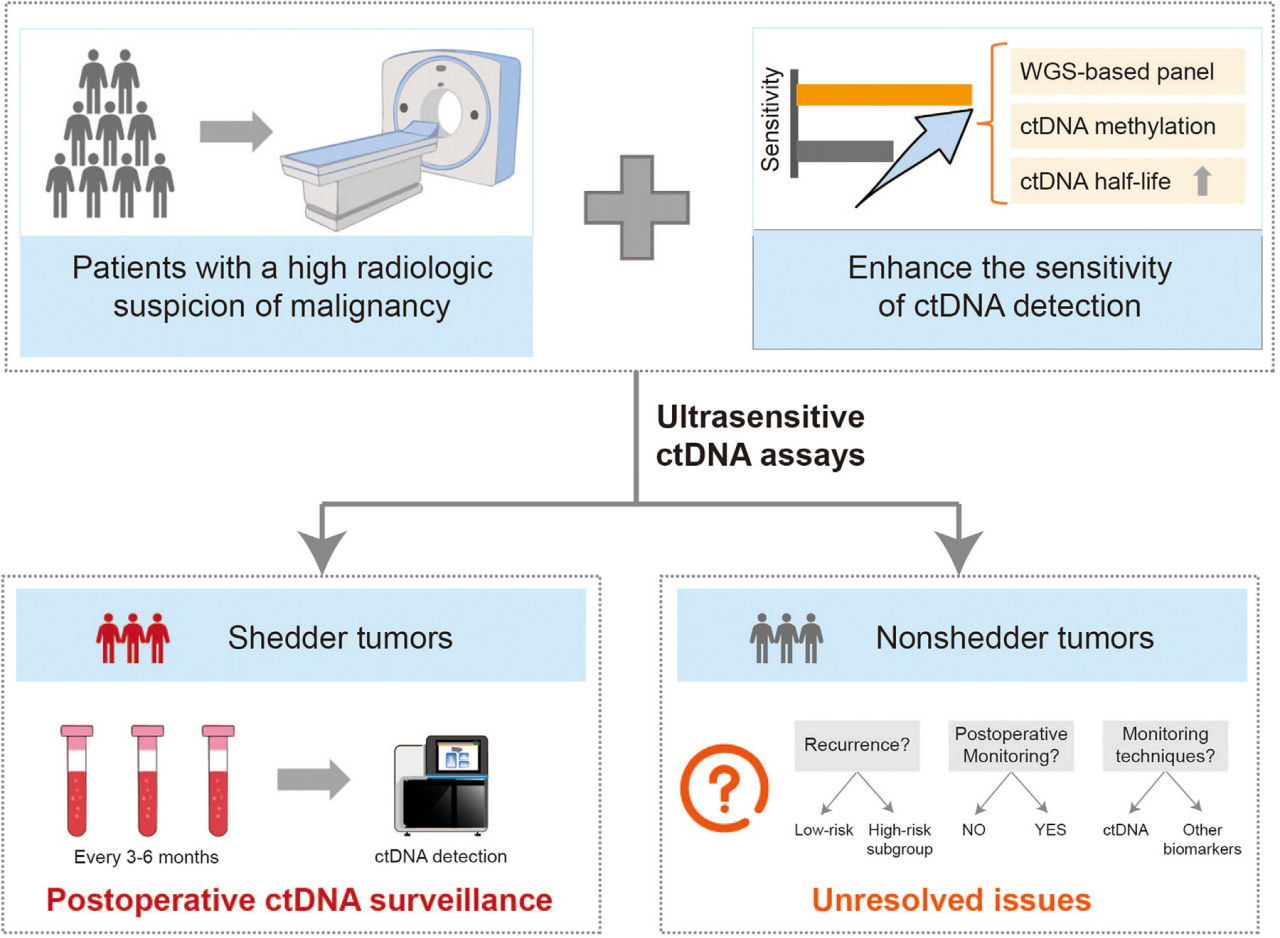
**Clinical prospects of nonshedder tumors.** First, through technological innovation, the sensitivity of ctDNA detection technology can be improved, thereby identifying lowshedder tumors within the nonshedder category and reclassifying them as shedders. For shedder tumors, regular postoperative ctDNA surveillance should be performed. However, for nonshedder tumors, several unresolved questions remain: What is their prognosis? Should they undergo monitoring? If so, which technologies are most appropriate?

Current evidence predominantly suggests that nonshedder tumors exhibit better prognosis compared to shedder tumors. However, approximately 20% of nonshedder tumor patients still experience recurrence and metastasis [5,6,14]. Notably, the ultrasensitive NeXT Personal assay demonstrated 100% 5-year survival in nonshedder patients [30], suggesting that recurrent cases in nonshedder cohorts might represent false negatives due to technical limitations rather than true biological behavior. Nevertheless, conclusive evidence remains lacking. As detection sensitivity continues to improve, critical questions emerge regarding nonshedder tumor management (Fig. 3): If the prognosis of nonshedder tumors is generally favorable, can subsequent MRD monitoring be omitted? Conversely, if a subset of high-risk patients still exists among nonshedder tumors, does ctDNA testing retain clinical value for these individuals, and should alternative biomarkers be considered for surveillance? These unresolved issues underscore the need for further investigation into the intrinsic prognostic characteristics of nonshedder tumors to guide personalized monitoring strategies.

## 5 Conclusion

The phenomenon of nonshedder tumors presents both challenges and opportunities in the field of cancer recurrence monitoring and personalized treatment. While nonshedder tumors are associated with a better prognosis compared to shedder tumors, the risk of recurrence still exists, necessitating a deeper understanding of the underlying mechanisms. Advances in ctDNA detection technologies allow us to detect much smaller amounts of ctDNA, avoiding the possibility of false negatives and thus identifying true nonshedder tumors for subsequent mechanistic exploration. Furthermore, the application of multi-omics approaches is crucial for unraveling the complex biological pathways that regulate ctDNA shedding. By enhancing our understanding of nonshedder tumor characteristics and refining prognostic stratification, we can optimize perioperative management, improve recurrence monitoring, and tailor personalized therapeutic strategies, ultimately enhancing patient outcomes.

Building on these research directions, our team has undertaken considerable investigation. We were among the first to systematically compare the detection performance of tumor-informed personalized panels, tumor-informed fixed panels, and tumor-agnostic fixed panels [5]. Furthermore, we developed ctDNA methylation-based assays and multi-omics methodologies integrating mutational and fragmentomic profiles to significantly enhance detection sensitivity [35,48]. Mechanistically, we identified a strong correlation between TP53 mutations and ctDNA shedding [5]. Future work will focus on elucidating the molecular mechanisms underlying nonshedder tumors and refining ctDNA detection systems to enhance the identification of high-risk patients and support clinical decision-making. We encourage further collaborative research to advance these efforts.

## CRediT authorship contribution statement

**Zifan Li:** Writing – review & editing, Writing – original draft, Visualization, Methodology, Investigation, Data curation, Conceptualization. **Yue He:** Writing – review & editing, Writing – original draft, Investigation. **Jing Bai:** Writing – review & editing, Writing – original draft, Investigation, Conceptualization. **Hongke Wang:** Writing – review & editing, Writing – original draft, Investigation. **Kezhong Chen:** Writing – review & editing, Writing – original draft, Supervision, Project administration, Funding acquisition, Conceptualization.
